# Experiences of Dissensus Between People Living With Mental Illness and Their Care Providers

**DOI:** 10.7759/cureus.101520

**Published:** 2026-01-14

**Authors:** Yuji Fujimoto, Takaomi Furuno, Narumi Fujino

**Affiliations:** 1 Department of Nursing, Faculty of Medicine, Saga University, Saga, JPN

**Keywords:** dissensus, mental illness, personal recovery, psychiatric care, shared decision making

## Abstract

Objective: Shared decision making (SDM) is fundamental to personal recovery; however, the values held by service users and care providers often differ. This study aimed to focus on dissensus, defined as a state in which value- and opinion-based differences between service users and care providers are not resolved but are maintained according to specific contexts, and to clarify the experiences through which service users recognized dissensus with care providers in psychiatric care settings.

Methods: Focus group interviews (FGIs) were conducted with six service users with prior experience of receiving psychiatric treatment or services. The data were analyzed using thematic analysis.

Results: Two major themes were identified as experiences of dissensus: the perception that clinical recovery was prioritized over personal recovery, and the feeling that opportunities for self-disclosure were constrained by the superior hierarchical positioning of care providers.

Discussion: The findings highlight a gap between the values of service users, who prioritize opportunities for challenge, autonomy, and identity reconstruction within equitable and collaborative relationships, and those of care providers, who prioritize safety, risk management, and role-based professional responsibilities. These dynamics suggest the importance of supporting collaborative partnerships that reconcile conflicting values in psychiatric care.

Conclusion: In the context of SDM for psychiatric care, the goal should extend beyond achieving consensus; explicitly acknowledging value conflicts arising from differing roles and fostering dissensus grounded in mutual respect may contribute to a more nuanced and effective approach to SDM.

## Introduction

Personal recovery, defined as the process of finding new meaning and purpose in life, has become the foundation of contemporary mental health policies [1]. In a semi-structured interview study of 12 psychiatric outpatients, Yüksel et al. [2] conceptualized personal recovery as “my journey,” emphasizing it as a multidimensional process of change and identity construction. Through this journey, service users develop unique identities and values. Conversely, care providers shape their identities and values based on clinical experiences, personal life histories, and professional backgrounds [3]. Given these different viewpoints, achieving complete alignment of values is inherently challenging [4]. Similarly, a qualitative study by Burger [5] indicated that disagreements and conflicts may arise within the collaborative recovery processes, potentially leading to negative interactions.

Shared decision making (SDM), which emphasizes information sharing between service users and care providers, is considered a core component of recovery-oriented systems [6]. Service users express a desire to receive comprehensive information and participate actively in decisions regarding their care [7]. However, they frequently report that their involvement in decision-making falls short of their preferences and perceived capabilities [8], resulting in deviations from the SDM principles [9]. In SDM within the field of mental health, service users feel that their experiential expertise is not recognized by care providers, a form of provider-held stigma that acts as a barrier to the expression of service users’ values and opinions [10].

To address such value conflicts, Value-Based Practice (VBP) has been proposed. VBP is a process that focuses on the often complex and competing diverse values involved and enables decision-making while maintaining a balance between the values held by each party [11]. In VBP, the concept of dissensus is employed as an idea that plays an important role in the values held by each party. Dissensus refers to a state in which value- and opinion-based differences that arise between service users and care providers are not resolved but rather are maintained while adapting to specific contexts [11]. In dissensus, the concept does not simply refer to a lack of agreement; rather, it assumes diversity of values and requires that mutual respect be maintained. In instances of dissensus, care providers are expected to respect the service user’s “my journey,” and service users are likewise expected to acknowledge the professional development and expertise of care providers. Although value conflict has traditionally been regarded as a barrier to SDM, exploring best practices for addressing dissensus may help overcome challenges in providing ethical support and implementing SDM. Therefore, this study aimed to clarify the experiences through which service users recognized dissensus with care providers, using semi-structured focus group interviews (FGIs).

## Materials and methods

Study design

This study aimed to clarify the experiences through which service users recognized dissensus with care providers in psychiatric care settings and adopted a qualitative research design using FGIs. FGIs are a method in which participants with shared experiences or backgrounds gather and, under the facilitation of a moderator, discuss a specific topic, thereby enabling the collection of rich data through group dynamics and interactions [12]. The dissensus examined in this study involves disagreements and value conflicts within relationships with care providers, and it was anticipated that such experiences might not be fully articulated through recall-based individual interviews. In contrast, FGIs provide a psychologically safe environment by bringing together individuals with shared experiences, enabling participants to recall latent experiences stimulated by the narratives of others. For these reasons, FGIs were considered the most appropriate method for capturing the essence of dissensus from the perspective of service users. FGIs have also been widely used in qualitative studies involving service users in psychiatric settings [13].

Participants

Participants were service users who had received mental health services, without restrictions on diagnosis or type of facility. The inclusion criteria for participants were being 20 years of age or older, having been diagnosed with a mental illness, having experience with inpatient or outpatient psychiatric treatment, and being able to provide informed consent to participate in the study as a service user. The exclusion criteria were patients who were hospitalized due to acute symptoms and service users who were receiving only psychological services, such as counseling, without seeing a physician.

Recruitment

A purposive sampling strategy was used to recruit participants who could provide rich data relevant to the research topic.

Recruitment was conducted through peer support groups, recovery college networks, and Wellness Recovery Action Plan facilitators. Snowball sampling was also performed by asking initial participants to introduce acquaintances engaged in similar activities. Following recommendations for small, interactive groups [12], two FGIs were conducted, each comprising three participants. Before participation, detailed explanations were provided regarding the study's purpose, the voluntary nature of participation, privacy protections, and potential burdens.

Data collection

Before each FGI, participants introduced themselves, and any reasonable accommodations for participation were confirmed (e.g., the need for breaks, permission to leave if feeling unwell, preferred seating). The flow of the FGI was then explained, after which participants completed a self-administered face sheet to provide information on gender, age group, hospitalization experience, and diagnosis. Next, in addition to explaining the term “dissensus” [11], we briefly described episodes related to psychiatric treatment, medication, and end-of-life care to help participants recall situations in which dissensus had occurred with their care providers [14]. After that, service users were asked to briefly describe what they had recalled regarding dissensus, and, after service users and researchers had established a shared understanding of dissensus, the FGIs were initiated to ensure the validity of the interview guide. The FGI was conducted by a single moderator with expertise in psychiatric nursing, and no facilitator was assigned. To promote open discussion through participant interaction, no strict time limits were imposed on individual speaking time; however, the moderator ensured that all participants had sufficient opportunity to express their views. An introductory segment of approximately 20-25 minutes was provided before beginning each FGI, and each participant took part only once. In the FGIs, participants were invited to describe situations in which they experienced dissensus with care providers, including the associated feelings and contextual factors. Examples of the interview questions included “Based on your past experiences, can you describe a specific situation in which you felt that your thoughts or perspectives differed from those of others?” and “How did you feel in that situation?” Using five questions, we asked about participants’ experiences of dissensus, as well as their emotions, interpretations, and actions in those moments. The interview guide was developed through discussions among multiple researchers with experience in dissensus-related literature, psychiatric nursing, and qualitative research, and was refined through repeated deliberations regarding the clarity and validity of the questions (Appendix A). The interviews were audio-recorded and transcribed verbatim. Data collection was conducted solely through FGIs, and no individual interviews were performed. Data were collected between May and June of 2023.

Data analysis

In this study, we conducted a thematic analysis using an inductive approach [15]. Thematic analysis is a descriptive approach that focuses on lived experience and is a qualitative analytic method used to understand the essence of the phenomenon under investigation. In this study, because the aim was to clarify service users’ experiences of dissensus with care providers from the service users’ perspective, and because the method emphasizes the meaning attributed to those experiences, thematic analysis was employed.

Transcripts were read repeatedly to ensure familiarity with the data. Meaningful units related to dissensus were coded, and similar codes were grouped into themes based on underlying patterns. Relationships among themes were examined and refined as needed. Credibility was enhanced through repeated discussions among three experts in psychiatric nursing research.

Ethical considerations

This study was conducted at the Department of Nursing, Faculty of Medicine, Saga University in Saga, Japan. This study was approved by the Ethics Committee of the Saga University Faculty of Medicine (Approval No. R5-2). Participants were informed twice, first through the recruitment document and again before the interview, about the purpose of the study, voluntary participation, anonymity, data handling procedures, and potential risks. Written informed consent was obtained from all participants.

## Results

Participant characteristics

Six individuals participated in the study (five men and one woman). Participants were in their 30s (n = 1), 40s (n = 2), and 50s (n = 3). Diagnoses included schizophrenia (n = 3), bipolar disorder (n = 2), and depression (n = 1). The mean number of hospitalizations was 2.5 (range: 1-8). The mean duration of each FGI was 77 minutes and 23 seconds.

Themes of dissensus experienced between service users and care providers

Two major themes were identified (Table 1). A-F in the table indicate participant identifiers.

**Table 1:** Experience of Dissensus Perceived by Service Users in Their Interactions With Care Providers

Major Theme	Subtheme	Code
Feeling that clinical recovery was prioritized over personal recovery	Desire to pursue challenges, such as employment, was overridden by recommendations prioritizing risk management and symptom exacerbation	B: Although I wanted to work after discharge, I continued attending the day-care program recommended by the hospital without feeling convinced.
		C: I found a sense of purpose at the vocational workshop and expressed my desire to quit day care and increase my workshop attendance, but this was not approved.
		A: When others, including the facility director, acknowledged my efforts and I felt motivated to try harder, I felt ashamed and angry when my situation was nevertheless attributed to my illness.
		A: When I consulted the day-care staff about the director’s suggestion that I temporarily attend the workshop, they did not approve it due to concerns about symptom worsening.
		A: Even though fluctuations in my mood are something only I can truly understand, I felt that my options were taken away simply because of my illness.
		A: It was difficult to negotiate with the day-care staff about the workshop, and I ultimately ended up going to day care by giving in to them.
		B: Supporters assumed that employment meant working full-time, so my desire to live on the income I could earn within my own capabilities was not understood.
		A: My statements were repeatedly dismissed as delusions, and I wished they would trust me, a patient who was anxious and struggling.
	Uniform, provider-driven recommendations were made regardless of symptoms or abilities	D: I wanted to decide on my treatment through mutual agreement based on advice from the medical staff, but the process became one-sided.
		D: The provider dominated the interaction, making it one-directional, and I could not fully express my own reasons or perspectives.
		F: During medication counseling, the provider spoke as if their view of what support I needed was the only correct one, making me feel as though no other options existed.
		F: In day care, I was made to do activities that did not interest me, and I questioned why I had to comply.
		D: I was not taught the purpose of occupational therapy or how it contributed to treatment; I simply made things because I was told to.
		E: I wanted to participate in sports at day care, but because it was my first day, I was made to play cards instead.
		D: Partly due to my lack of knowledge, I simply followed what I was told, without any choice or advocacy for my rights.
		E: Even though I said I was too afraid to sleep in my room because of fear, I was sent back to my room from the day room at every staff round.
		D: Because I saw myself as a patient, I did not express my own wishes or hopes, and was unable to manage myself.
		D: I remained under strict control and was not given explanations in many situations.
	Providers tried to reduce my burden, even though I wanted to manage things on my own	F: My statement that I could manage things on my own was not accepted, and I was told that my parents’ opinions were also important, which I could not agree with.
		D：I believed I was capable of managing myself, but I was not allowed to do so.
		C: Because people with mental illness are often hurt or misunderstood, I feel that in psychiatry, many patients avoid speaking honestly.
		F: My autonomy was not acknowledged.
Opportunities for self-disclosure were constrained by the superiority of care providers	Behaviors were dismissed as symptoms, and participants felt they were not given adequate opportunities to assert themselves	A: When I reported conflict within my family, it was dismissed as a symptom-driven misunderstanding, and I was given an injection.
		B: There were times when, if I said “I’m fine,” providers suspected that something must be wrong.
		A: The more I insisted that I was not feeling unwell, the more providers doubted my symptoms, and suppressing my true feelings to avoid misunderstanding undermined my ability to make decisions.
		A: Because I tried so hard to explain, the doctor assumed it was delusional thinking, and due to my status as a psychiatric patient, I had no way to prove that what I said was true.
		F: I wanted them to focus on my feelings and think things through with me, but they prioritized my family’s opinions.
		A: My mother’s opinions were respected because providers were concerned about the conflict I faced at home.
		F: On the day of my appointment, I asked what had been discussed with my family, but I was not told, and I could not understand the reason.
	Distrust toward care providers reduced opportunities for honest communication	E: I felt that providers did not listen to the feelings behind my words and only tried to solve the problem, so I closed myself off.
		D: The nurse did not ask about what I truly wanted to express and instead asserted the nurse’s own perspective.
		B: I can talk to familiar providers as one person to another—not as a patient—but I cannot talk openly to those who seem to treat everything merely as part of their job.
		A: When I was younger, I brought up many things with my doctor, but I realized that I could talk only when I felt trust in the doctor.
		F: Not having my feelings acknowledged made me feel as though something that had once opened within me had closed again.
		C: Even with doctors, if there is no sense of trust at a human level, I cannot speak openly; when I do feel trust, I sometimes talk more to other healthcare professionals than to my primary doctor.
	Feeling that providers held power in treatment decisions made participants reluctant to speak honestly	C: In psychiatric settings, patients feel they must be obedient out of fear that resisting or displeasing providers could lead to hospitalization.
		A: To maintain the relationship with providers, I sometimes watched their reactions, told harmless lies, or pretended to be fine.
		F：The primary doctor held full control, and I felt I was not in a position or relationship where I could express my thoughts, so I said nothing.
		B：Even when I felt the hospital acted for its own convenience, I considered possible harm or disadvantage to myself and my future care, watched the provider’s reactions, and ultimately gave in.
		A: In the doctor–patient relationship, I often pretended to understand or acted agreeable, and avoided saying things that did not need to be said.
		D: Because my primary doctor was the hospital director, I could not raise concerns; when I voiced dissatisfaction to a nurse, I was forced to discharge.
		B: I felt I could not move forward with what I wanted to do unless I acted subservient, so even when I could not sleep, I said that I had slept.
		B: Whenever hospitalization was mentioned, I felt discouraged and began watching the provider’s reactions.
		C: If doctors or nurses developed a negative impression of me, I feared they would increase medication or recommend hospitalization, so even when I felt unwell, I downplayed my symptoms.
		A: I felt that depending on how I responded, I might be hospitalized, and sometimes the relationship between mental health providers and people with mental illness felt difficult to navigate.
	Providers assumed diminished understanding due to symptoms and withheld desired information	B: When medications were adjusted, I wanted explanations about side effects, but sometimes I was only told that the dose would be increased.
		F: Even though medications caused physical discomfort, I was not given clear explanations and did not feel I was in a position to ask for them.
		B: During hospitalization, I received insufficient explanation, and I could not accept that belts and shoelaces were taken from me or that I was placed in a seclusion room for six months.
		B: I wanted an explanation of risks and how dangerous items would be managed, but without such an explanation and being forced to comply, I found it difficult to trust the providers.
		F: When I brought in a laptop, it was kept at the nurse station and returned only when I left, but I was never given a convincing explanation, which left me confused.
		D: Even though I was taking the prescribed medication, my condition worsened, and I felt as if my body was being controlled by medications prescribed without my knowledge.
		A: Providers tend to attribute things to illness or explain them with softened expressions, but I feel that being told things directly would help me understand and accept them.
	Participants wanted their needs to be respected, but rules and institutional policies were prioritized	F: In day care, I was not allowed to go to the hospital shop or freely go outside.
		F: I had no interest in the day-care program, and when I said I wanted to use my laptop, I was told that it was not a place where laptops were allowed.
		E: Looking back, I realized that absolute rules had been ingrained in me without my noticing.
		B: When I asked nurses for the reason, they only said “It’s the rule,” and they took away my earphones and phone; I felt ashamed and deprived of freedom.

Feeling that clinical recovery was prioritized over personal recovery. The desire to pursue challenges, such as employment, was overridden by recommendations prioritizing risk management and symptom exacerbation.

Participants described that when they expressed motivation to work or engage in new activities, care providers often restricted their efforts due to concerns about symptom deterioration or risk management. Service users attempted to pursue their goals with an understanding of their condition; however, they encountered situations in which care providers' judgments constrained their aspirations. 

“When the director of a workshop invited me to attend temporarily, I consulted the day-care staff, but they refused due to concerns about symptom worsening," said one service user.

Another service user said, “Even though only I can understand my own emotional ups and downs, I felt my options were taken away simply because of my illness.” 

“I wanted to work after discharge, but I ended up attending a day care recommended by the hospital, even though I was not convinced,” said another service user.

Uniform, provider-driven recommendations were made regardless of symptoms or abilities. Participants reported instances in which their preferences were not respected, with care providers unilaterally directing decisions regarding activities, treatment plans, or day-care attendance. These accounts reflect limited opportunities for autonomous decision-making.

“I wanted to decide my treatment plan in a mutually agreeable way based on advice from the providers, but it ended up being one-sided,” was the view of one service user.

Another service user said, “In day care, I was made to do activities I had no interest in, and I questioned why I had to comply.”

"Providers tried to reduce my burden, even though I wanted to manage things on my own," said a service user.

Participants felt capable of managing their own care; however, they perceived that care providers assumed the need to reduce their burden and consequently imposed restrictive measures. These experiences were interpreted as signaling a lack of confidence in their self-management abilities, thereby limiting opportunities for personal growth.

One service user said this: “I believed I could manage myself, but I wasn’t allowed to."

Another service user said, “My statement that I could handle things on my own wasn’t acknowledged. I was told that my parents’ opinions were also important, and I couldn’t accept that."

The superior hierarchical positioning of care providers constrained opportunities for self-disclosure. Behaviors were dismissed as symptoms, making participants feel they were given limited opportunities to assert themselves. Participants reported instances in which issues shared, such as family conflicts or changes in physical condition, were dismissed as delusions or symptoms of mental illness rather than acknowledged as factual information. Their experiences conveyed a sense that their thoughts were automatically interpreted through the lens of illness.

“When I talked about conflict at home, they didn’t believe me and said it was a symptom-driven misunderstanding. They gave me an injection,” said a service user.

“Even when I said I wasn’t feeling unwell, providers often doubted my words as symptoms. I suppressed my real feelings to avoid misunderstanding, which undermined my ability to make decisions,” said another service user.

Distrust toward care providers reduced opportunities for honest communication. Participants felt that some providers engaged in mechanical, role-driven interactions without considering their emotions or values. This dynamic led them to believe that honest communication was impossible.

“With providers I trust, I can talk as one person to another, not as a patient. But I cannot talk openly to providers who seem to treat everything as just part of their job,” opined one service user.

Another service user said, “Care providers didn’t listen to the feelings behind my words; they just tried to solve the problem. Eventually, I closed myself off.” 

Feeling that providers held power in treatment decisions made participants reluctant to speak honestly. Participants described that concerns about increased medication or the possibility of hospitalization made it challenging to express their true thoughts or symptoms. This perceived power imbalance in the care relationship discouraged open communication.

“If doctors or nurses got a bad impression of me, I feared they would increase my medication or recommend hospitalization, so even when I felt unwell, I downplayed my symptoms,” were the words of one service user.

Another service user said, “Whenever hospitalization was mentioned, I felt discouraged and began watching the providers’ reactions.”

“To maintain a good relationship with providers, I sometimes watched their expressions, told small lies, or pretended to be fine,” said another service user.

Providers assumed diminished understanding owing to symptoms and withheld desired information. Participants described that decisions regarding medication or treatment were often made without sufficient explanation. They felt that providers assumed their understanding was impaired by symptoms, creating a sense of opacity in the decision-making process.

One service user said, “When medications were adjusted, I wanted explanations about side effects, but sometimes they only told me that the dose was being increased.”

Another service user said, “I wanted them to explain the risks and how they would ensure my safety regarding dangerous items, but they gave no explanation, and following their orders felt unacceptable.”

“When I brought in a laptop, it was kept at the nurse’s station and returned when I left, without any convincing explanation. I felt confused,” said another service user.

Participants wanted their needs to be respected, but rules and institutional policies were prioritized. Participants reported that institutional rules were often prioritized over their personal needs and preferences, thereby constraining their daily activities.

One service user said, “In day care, I couldn’t go to the shop, to the hospital, or freely go outside.”

“Looking back, I realized that absolute rules had been imposed on me without my noticing,” said another service user.

## Discussion

This study identified two major themes characterizing dissensus between service users and care providers: the perception that clinical recovery was prioritized over personal recovery, and a sense of constraint in self-disclosure owing to the hierarchical superiority of care providers. These findings indicate a gap between service users’ values, such as autonomy, the desire for challenges, and identity development within collaborative relationships, and care providers’ values, including safety, risk management, and professional responsibility [16, 17]. Dissensus does not reject value conflict; rather, it presupposes mutual respect in the absence of a single correct viewpoint [18]. The conflicts observed in this study reflect the contrasting value systems shaped by lived versus professional experiences. In recovery-oriented practice, tensions between autonomy and risk management are common [19], and the themes identified in this study underscore the central challenge of reconciling these opposing values in psychiatric care. Speyer et al. [20], drawing on the perspectives of researchers with lived experience, propose reframing dissensus not as a problem to be resolved but as a source of innovation. Exploring dissensus within the reciprocal relationship between service users and care providers can therefore be seen as having the potential to promote more collaborative and ethical decision-making.

VBP emphasizes the importance of openly acknowledging dissensus to build authentic partnerships. However, participants reported that the superior position of care providers limited their capacity for self-disclosure, an experience that contradicts VBP principles and reflects ongoing stigma and rigid clinical assumptions in psychiatric care [10]. Furthermore, VBP suggests that making dissensus explicit is often difficult in real-world practice, and our findings suggest that this difficulty persists in psychiatric settings. Although SDM emphasizes patient-centeredness, it does not always imply patient-led care; under certain conditions, provider-led approaches may be more ethically appropriate and clinically effective [21]. However, from the perspective of service users, strongly provider-led approaches may appear to conflict with the principle of patient-centeredness, underscoring the need to distinguish provider-led care from genuinely patient-led care.

The themes of dissensus experienced by service users in this study are consistent with recent research that has highlighted issues such as disagreements within recovery-oriented collaborative processes and the dominance of care providers [5, 10]. These findings suggest inherent characteristics of dissensus within psychiatric care.

Implications for practice

Service users perceived that care providers tended to prioritize safety and risk avoidance when they expressed a desire to take on new challenges. Rather than seeking consensus solely for the purpose of risk avoidance, care providers should adopt a patient-centered perspective that regards such situations as “opportunities for growth,” even when they involve responsibility or effort on the part of service users. It is important for care providers and service users to share the risks and responsibilities associated with these challenges while maintaining a balance between their respective values. Service users also reported feeling unrecognized as equal partners in decision-making [22]. Dissensus is a practice grounded in mutual respect, and when this mutual respect is undermined, it can function not as constructive dialogue but as a form of oppression. The findings of this study provide important practical implications, underscoring the need for care providers to adopt a reflective stance toward their relationships with service users. Subtle value conflicts may easily go unnoticed, and assuming shared values can lead to unintended harm [23]. Therefore, establishing collaborative partnerships requires making implicit value gaps explicit, confirming the user’s lived values, and fostering mutual respect while integrating professional perspectives. In particular, when value conflicts have not yet emerged, it is crucial to carefully explore service users’ experience-based values and clarify how these differ from care providers' professional values.

Strengths and limitations

A strength of this study lies in its focus on dissensus from the perspective of service users rather than consensus. In recent years, the lived experience of service users has been increasingly emphasized not only as a source of knowledge that cannot be captured by care providers but also as a central component of treatment and mental health policy [24]. In light of this trend, elucidating experiences of dissensus from the perspective of service users provides foundational insights that can contribute to rethinking new possibilities for SDM and recovery-oriented decision support. Moreover, SDM involves information sharing and collaborative consensus building [25]; however, disagreements are often perceived as problematic. A qualitative meta-summary by Mertens et al. [10] revealed that the primary barrier to SDM is not the presence of disagreement itself, but rather the absence of being treated as a person of equal worth. The concept of dissensus offers a complementary framework by focusing on mutual respect for differing values. Future research should aim to develop objective indicators for evaluating how well value conflicts are recognized and addressed in practice.

This study has some limitations. First, the lived experiences of service users change over time and across situations [24], and the themes identified in this study reflect their experiences at a specific point in time. Therefore, the findings of this study require cautious interpretation, and future longitudinal or follow-up studies are needed to deepen understanding by capturing temporal changes. Additionally, this study aimed to gain an in-depth understanding of dissensus experiences from service users’ narratives, and data saturation was considered to have been reached based on the recurrence and consistency of themes. However, because the number of participants was small (n = 6), the generalizability of the findings remains limited. In addition, in this study, snowball sampling was used with the highest priority placed on participants’ psychological safety; however, there was an imbalance in the gender distribution of the participants, and it is possible that gender diversity was not sufficiently represented. Furthermore, the findings reflect the subjective perceptions of service users; care providers may have intended to support personal recovery or encourage self-disclosure; however, communication mismatches may have occurred. Future research should examine dissensus from the perspective of care providers to identify best practices for managing value conflicts in psychiatric care.

## Conclusions

This study elucidated how service users experience dissensus with care providers in psychiatric care settings. A key finding was the perceived gap between service users’ pursuit of autonomy, challenge, and identity reconstruction, and care providers’ prioritization of safety, risk management, and professional responsibilities. These findings underscore the importance of establishing collaborative partnerships capable of addressing conflicting values. In the context of SDM, the goal should not be consensus alone; explicitly acknowledging value conflicts and fostering dissensus grounded in mutual respect may support the development of a more effective SDM approach in psychiatric care.

## Figures and Tables

**Figure 1:**
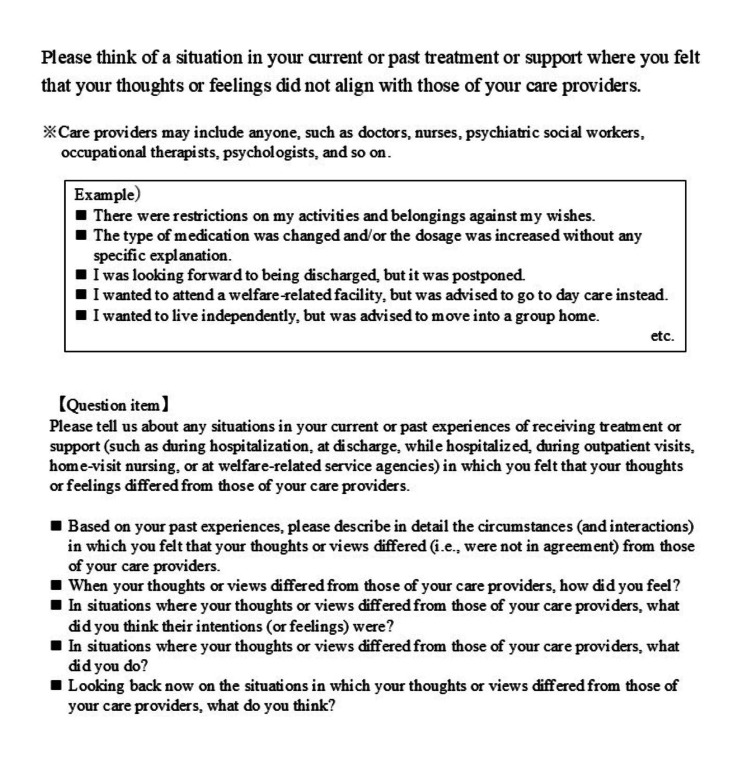
Interview Guide

## Appendices

Appendix A 

## References

[1] World Health Organization (2013). World Health Organization: Mental health action plan 2013-2030. Comprehensive Mental Health Action Plan 2013-2030.

[2] Yüksel R, Çekiç Y, Çolak B (2025). “My journey”: a qualitative study of recovery from the perspective of individuals with chronic mental illness. Int J Ment Health Nurs.

[3] Dudas RB (2021). Values-based practice: how can history taking help psychiatrists explore the values involved in clinical decision-making?. BJPsych Adv.

[4] Trujols J, Portella MJ, Iraurgi I, Campins MJ, Siñol N, de Los Cobos JP (2013). Patient-reported outcome measures: are they patient-generated, patient-centred or patient-valued?. J Ment Health.

[5] Burger TJ, van Eck RM, Lachmeijer M (2024). Perspective matters in recovery: the views of persons with severe mental illness, family and mental health professionals on collaboration during recovery, a qualitative study. BMC Psychiatry.

[6] Grim K, Rosenberg D, Svedberg P, Schön UK (2016). Shared decision-making in mental health care-a user perspective on decisional needs in community-based services. Int J Qual Stud Health Well-being.

[7] de la Espriella R (2020). Decision making in psychiatric patients: a qualitative study with focus groups. Rev Colomb Psiquiatr (Engl Ed).

[8] Haugom EW, Stensrud B, Beston G, Ruud T, Landheim AS (2022). Experiences of shared decision making among patients with psychotic disorders in Norway: a qualitative study. BMC Psychiatry.

[9] Matthias MS, Salyers MP, Rollins AL, Frankel RM (2012). Decision making in recovery-oriented mental health care. Psychiatr Rehabil J.

[10] Mertens L, Vandenberghe J, Bekkering G (2025). Navigating power imbalances and stigma in mental healthcare: patient-reported barriers and facilitators to participation in shared decision-making. Health Expect.

[11] Fulford KWM, Peile E, & Carroll H (2016). Essential Values-Based Practice: Clinical Stories Linking Science with People.

[12] Nyumba TO, Wilson K, Derrick CJ, Mukherjee N (2017). The use of focus group discussion methodology: insights from two decades of application in conservation. Methods Ecol Evol.

[13] Yamaguchi S, Shiozawa T, Matsunaga A (2020). Development and psychometric properties of a new brief scale for subjective personal agency (SPA-5) in people with schizophrenia. Epidemiol Psychiatr Sci.

[14] Fulford KW (2008). Values-based practice: a new partner to evidence-based practice and a first for psychiatry?. Mens Sana Monogr.

[15] Braun V, Clarke V (2006). Using thematic analysis in psychology. Qual Res Psychol.

[16] Leamy M, Bird V, Le Boutillier C, Williams J, Slade M (2011). Conceptual framework for personal recovery in mental health: systematic review and narrative synthesis. Br J Psychiatry.

[17] Croucher S, Williamson GR (2013). Risk assessment in mental health: introducing a traffic light system in a community mental health team. Open Nurs J.

[18] Plagnol A (2013). Psychiatry and values-based medicine (Article in French). Ann Med Psychol (Paris).

[19] Ørjasæter KB, Almvik A (2022). Challenges in adopting recovery-oriented practices in specialized mental health care: “How far should self-determination go; should one be allowed to perish?”. J Psychosoc Rehabil Ment Health.

[20] Speyer H, Ustrup M (2025). Embracing dissensus in lived experience research: the power of conflicting experiential knowledge. Lancet Psychiatry.

[21] Resnicow K, Catley D, Goggin K, Hawley S, Williams GC (2022). Shared decision making in health care: theoretical perspectives for why it works and for whom. Med Decis Making.

[22] Dahlqvist Jönsson P, Schön UK, Rosenberg D, Sandlund M, Svedberg P (2015). Service users' experiences of participation in decision making in mental health services. J Psychiatr Ment Health Nurs.

[23] Petrova M, Dale J, Fulford BK (2006). Values-based practice in primary care: easing the tensions between individual values, ethical principles and best evidence. Br J Gen Pract.

[24] Montague Cardoso K, Sunkel C, Burgess RA (2024). Elevating the voices of lived experience to combat structural barriers and improve mental health globally. PLOS Mental Health.

[25] Charles C, Gafni A, Whelan T (1997). Shared decision-making in the medical encounter: what does it mean?. Soc Sci Med.

